# Dosimetric comparison of incidental radiation to the internal mammary nodes after breast-conserving surgery using 3 techniques-inverse intensity-modulated radiotherapy, field-in-field intensity-modulated radiotherapy, and 3-dimensional conformal radiotherapy: A retrospective clinical study

**DOI:** 10.1097/MD.0000000000017549

**Published:** 2019-10-11

**Authors:** Yuanfang Song, Ting Yu, Wei Wang, Jianbin Li, Tao Sun, Pengfei Qiu, Min Xu, Qian Shao

**Affiliations:** a Department of Radiation Oncology, Shandong Cancer Hospital and Institute, Shandong First Medical University and Shandong Academy of Medical Sciences, Jinan; b Tianjin Medical University, Tianjin Medical University Cancer Institute and Hospital, National Clinical Research Center for Cancer, Key Laboratory of Cancer Prevention and Therapy, Tianjin's Clinical Research Center for Cancer, Tianjin; c Breast Cancer Center, Shandong Cancer Hospital and Institute, Shandong First Medical University and Shandong Academy of Medical Sciences, Jinan, China.

**Keywords:** breast-conserving postoperative radiotherapy, incidental irradiation dose, internal mammary node region, treatment planning comparison

## Abstract

**Background:**

The study aimed to evaluate and compare the dosimetric parameters of incidental irradiation to internal mammary node (IMN) from inverse intensity-modulated radiotherapy (I-IMRT) and field-in-field IMRT (F-IMRT), and 3-dimensional conformal radiotherapy (3D-CRT) in patients after breast-conservation surgery (BCS).

**Methods:**

Eighty-four patients with BCS were selected. The breast, tumor bed, and IMN, including intercostal spaces (ICS) 1 to 3, were contoured. Three plans were generated. The prescription doses for the breast and tumor bed were 50.4 Gy/28 F and 60.2 Gy/28 F, respectively. If there was no tumor bed boost, patient was treated with 50 Gy/25 F for the whole breast only. The IMN was not included in planning target volume.

**Results:**

The median mean dose (D_mean_) of the IMN_total_ (ICS 1–3) was 2740.2 cGy, 2973.9 cGy, and 2951.4 cGy for I-IMRT, F-IMRT, and 3D-CRT, respectively. Differences were not detected between any of the plans. After separating ICS 1 to 3 for further analysis, neither of the D_mean_ of ICS 1 to 2 was significantly different between the plans. However, for ICS 3, the median D_mean_ was highest for I-IMRT, and those for 3D-CRT and F-IMRT were not significantly different. After separating the 3 techniques for further analysis, the median D_mean_ was highest in ICS 3 and lowest in ICS 1 for all the 3 techniques.

**Conclusion:**

All 3 techniques failed to attain an adequate dose to cure subclinical disease, and there were no significant differences among the 3 techniques. It is risky to avoid IMN irradiation (IMNI) using any of the 3 techniques during whole-breast radiotherapy in women with indications for elective IMNI. However, in era of systematic therapy, whether the incidental dose could meet clinical acquirements needs further follow-up.

## 1 Introduction

With the wide acceptance of breast-conservation surgery (BCS) for early-stage breast cancer patients, whole-breast radiotherapy (WBRT) has become an indispensable part of systematic therapy based on studies showing that BCS + WBRT reduces local recurrence and improves survival.^[1,2]^ Recently, the MA.20^[3]^ and European organization for research and treatment of Cancer^[4]^ trials indicated that the addition of regional nodal irradiation reduced the rate of breast-cancer recurrence, but the overall survival (OS) was not improved. The 2 studies failed to demonstrate whether the benefit could be attributed specifically to internal mammary node (IMN) inclusion because other lymph node regions were also included in the radiotherapy group. Furthermore, the Danish Breast Cancer Cooperative group-Internal mammary node ^[5]^ study showed significant improvements in OS for the radiotherapy group. However, because patients with left-sided breast cancer did not undergo internal mammary node irradiation (IMNI), the potential radiation-induced cardiotoxicity was unclear. As a critical pathway, the internal mammary chain is as important as the axillary chains. Although IMNI can control or eliminate tumor cells in this region, several studies have shown that IMNI causes an increase in cardiac deaths due to exposure of the heart to increased radiation.^[6–8]^ Furthermore, despite the high IMN involvement by IMN dissection and internal mammary sentinel lymph node biopsy of early breast cancers,^[9–11]^ the IMN recurrence rate is low when patients are treated with standard tangential irradiation.^[12]^ Consequently, whether the IMN should be included in the target volume remains controversial.

The IMN can be exposed to incidental irradiation due to its proximity to the treatment fields (whole breast or chest wall).^[13,14]^ Hare et al^[14]^ found that 73% of patients in their study had complete or partial inclusion of the IMN with standard tangents when the IMN was not an irradiated target. Therefore, it is possible to assume that the low risk recurrence of IMN may be attributed to the incidental dose in this region.

Several studies pertaining to incidental irradiation of the IMN have shown that the incidental dose is inadequate for sterilizing subclinical disease in the untargeted region. Nevertheless, the technique used in these studies was 3-dimensional conformal radiotherapy (3D-CRT), which was not adopted as routinely at the time as intensity-modulated radiotherapy (IMRT). Therefore, in this study, we examined incidental IMN dose coverage with inverse intensity-modulated radiotherapy (I-IMRT), field-in-field (FIF) intensity-modulated radiotherapy (F-IMRT), and 3D-CRT, and compared the 3 techniques.

## 2 Materials and methods

### 2.1 Patients

Women with BCS who underwent radiotherapy were selected. The internal mammary and supraclavicular lymph nodes were not included in the planning target. Ineligibility criteria included bilateral breast cancer and patients who required IMNI in the treatment target volume. The internal mammary nodes were negative when evaluated with enhanced magnetic resonance imaging before surgery by a breast radiologist. This study was approved by the Institutional Review Board (Shandong Tumor Hospital Ethics Committee). Because this was a retrospective study, the written informed consent was not obtained.

### 2.2 Computed tomography simulation

All patients were immobilized in a supine position on a breast board with both arms fully abducted (90 degrees or greater) and externally rotated. Planning computed tomography (CT) was obtained with 3-mm-thick sections and was extended from the chin to the upper abdomen without enhanced contrast.

### 2.3 Target delineation and dose prescription

Volume delineation was performed on Eclipse, version 8.6 (Varian Medical Systems Inc., Palo Alto). The delineation of clinical target volume of the breast (CTVbreast) was based on the confines of the wire placed during simulation CT scanning. The medial, lateral, inferior, and superior borders of CTVbreast were identified based on wire markers and assisted by CT images to include all apparent breast tissue. Then, the borders of CTVbreast were adjusted according to the location of the tumor bed and the range of mammary gland shown by the simulation CT. The anterior was retracted 5 mm from the skin surface and limited posteriorly by the pectoralis muscles or pleura-rib junction. The planning target volume (PTVbreast) was created by expansion of a 5-mm margin in all directions to the CTVbreast, but was cropped 5 mm away from the skin; overlapped lung tissues were also removed. The tumor bed was contoured based on surgery clips and/or seroma. The boost planning target volume (PTVtumor bed) was defined as the gross target volume with a 10-mm margin in all directions and was then retracted to within the PTVbreast. The IMN target volume was contoured by identification of the internal mammary vessels and a 5-mm CTV expansion was used in the anterior, posterior, and lateral directions. Then, the anterior direction was retracted to the posterior surface of the pectoralis or major muscle, and the posterior direction was retracted to the pleura. The target extended from the cranial aspect of the first rib to the cranial aspect of the fourth rib. IMN contouring was conducted by 1 radiation oncologist and verified by another. The OARs including the heart, lungs, and spinal cord were contoured.

For the patients with a tumor bed, the whole breast and tumor bed were treated with 1.8 and 2.15 Gy, respectively, in 28 fractions for a total dose of 50.4 and 60.2 Gy, respectively. For the patients who underwent radiation for the whole breast only, a common radiotherapy strategy of 50 Gy/25 F to the whole breast was adopted.

### 2.4 Treatment plan

For every patient, 3 radiotherapy plans were designed (I-IMRT, F-IMRT, and 3D-CRT) by 1 physicist using Eclipse, version 8.6 (Varian Medical Systems Inc.). For patients with a tumor boost, all 3 plans incorporated the simultaneous integrated boost (SIB) technique. The details of the 3 techniques are described below.

The I-IMRT plan was created using 6-MV photon beams, including 2 tangential fields with 2 to 4 additional fields. The field weights were all the same. Additionally, the tangential fields extended 2 cm anteriorly to the chest to provide coverage of the “flash” region. The planning acceptance criterion for the target dose coverage was 95% of the PTV (PTVbreast and PTVtumor boost) receiving the prescription dose. No constraint was given to the IMN. For IMRT optimization, a minimum segment area size of 9 cm^2^ and a minimum of 5 monitor units (MuS) were used. The total segments were limited to no more than 30. Additionally, all plans were optimized to minimize the normal tissue doses while achieving sufficient target coverage.

For F-IMRT, 6-MV photon beams were delivered to the whole breast, whereas the tumor boost was conducted using electrons. The electron ray energy was based on the depth of the tumor bed by adjustment. To remove the hot spots and improve homogeneity, we used the FIF technique based on 2 parallel-opposed tangential fields that covered the contoured target volume with Multi-leave collimators (MLC) blocks. Generally, 2 to 5 subfields were added in each direction. The weights of the 2 opposed tangential fields were equal or almost equal, and the weights of the different subfields were optimized manually to minimize the dose to the normal tissues. All plans were optimized to deliver at least 90% of the PTV receiving the prescription dose and to manipulate the MLCs to ensure that the maximum dose of the PTV areas would not exceed 107% of the isodose line.

The 3D-CRT plans used 2 opposed tangential beams alone with a block. No wedge was used. The weights of the 2 opposed tangential beams were adjusted to allocate the hot spots and deliver a homogeneous dose to the target volume. Accordingly, all plans were optimized to deliver at least 90% of the PTV receiving the prescription dose. The type of beams and electron ray energy were the same as described for the F-IMRT.

For these 3 treatment plans, the acceptance criteria for the normal tissues were as follows: less than 25% of the ipsilateral lung received ≥20 Gy, no more than 20% of the whole lungs received ≥20 Gy, the mean dose to the ipsilateral lung was ≤15 Gy, less than 15% of the contralateral lung received ≥20 Gy, no more than 15% of the heart received ≥10 Gy for left-sided tumors, and the mean dose to the heart was limited to ≤5 Gy. The maximum spinal cord dose was less than 45 Gy. No constraint was given to the IMN.

### 2.5 Dosimetric evaluation parameters and statistics

Dosimetric parameters were extracted from a dose–volume histogram. For the 3 intercostal spaces (ICS 1–3) we contoured, the mean dose (D_mean total_) of the IMN (IMN_total_) was calculated, and the per cent volumes of the IMN_total_ receiving 30, 40, and 50 Gy (Vx) were calculated. The mean dose (D_mean 1_), Vx, and volume were calculated for ICS 1. The above parameters for ICS 1 were also calculated for the second intercostal space (ICS 2) and the third intercostal space (ICS 3), respectively.

Statistical analysis was performed using the SPSS19.0 software (SPSS Inc. Chicago, IL). The data are represented as the median (range). The Wilcoxon Cox test was used because the distributions in the datasets were skewed. Differences were considered significant at *P* < .05.

## 3 Results

### 3.1 Patient characteristics

Eighty-four consecutive breast cancer patients who underwent radiotherapy after BCS, and were treated in Shandong Cancer Hospital affiliated to Shandong University from October, 2015 to January, 2017 were selected. Among them, 70 patients had tumor bed boosts and 14 patients received WBRT alone. The other clinical characteristics are shown in Table 1.

**Table 1:**
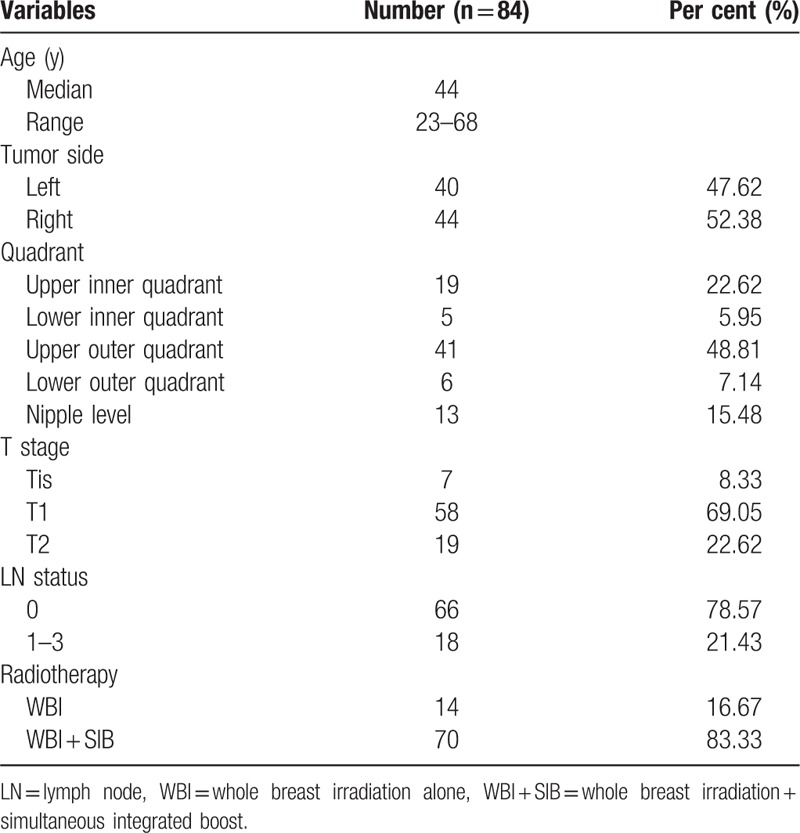
Clinical characteristics of the patients.

### 3.2 Dose parameters for the IMN_total_

The median D_mean_ for the IMN_total_ from the 3 techniques (I-IMRT, F-IMRT, and 3D-CRT) were 2740.2, 2973.9, and 2951.4 cGy, respectively, and no significant differences were found between any of the techniques. The V30 and V40 with I-IMRT were less than those with 3D-CRT or F-IMRT. The V50 was lowest for I-IMRT and highest for 3D-CRT (Table 2).

**Table 2:**
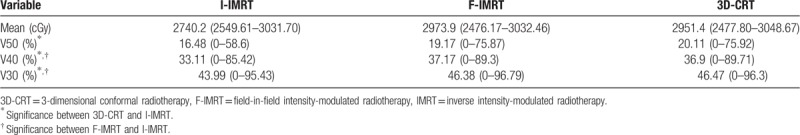
Comparison of dose coverage for the 3 intercostal spaces.

### 3.3 Dose parameters for the first 3 intercostal spaces separately

Regarding ICS 1, the median D_mean 1_ obtained with the 3 techniques (I-IMRT, F-IMRT, and 3D-CRT) were 1383.85, 1422, and 1228.1 cGy, respectively, and no significant differences were found between the techniques. The V30 was higher in 3D-CRT and F-IMRT than in I-IMRT, and the same results were obtained for the V50. The V40 was highest for F-IMRT, followed by 3D-CRT; I-IMRT was the lowest (3D-CRT vs F-IMRT; *P* = .026, I-IMRT vs 3D-CRT; *P* < .001, and I-IMRT vs F-IMRT; *P* < .001) (Table 3).

**Table 3:**
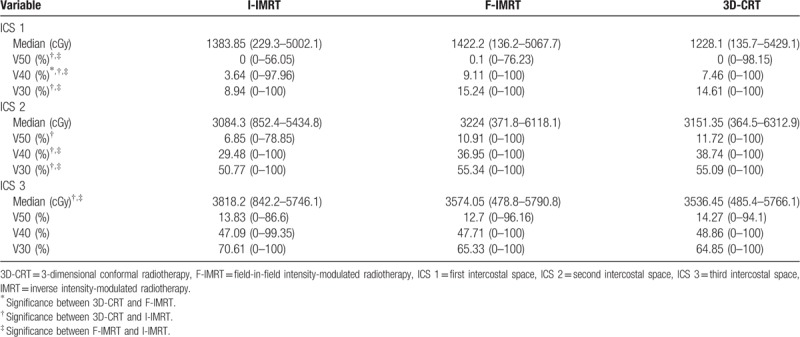
Comparison of dose coverage for 3 intercostal spaces separated.

Regarding the ICS2, the median D_mean 2_ obtained with the 3 techniques (I-IMRT, F-IMRT, and 3D-CRT) were 3084.30, 3224.00, and 3151.35 cGy, respectively. No significant differences were found between the techniques. For the V30 and V40, 3D-CRT and F-IMRT were higher than I-IMRT. The V50 was lowest for I-IMRT and highest for 3D-CRT (Table 3).

Regarding to the ICS 3, the median D_mean 3_ obtained with the 3 techniques (I-IMRT, F-IMRT, and 3D-CRT) were 3818.20, 3574.05, and 3536.45 cGy, respectively, with the value obtained for I-IMRT higher than the values obtained for F-IMRT and 3D-CRT; however, the median values obtained for F-IMRT and 3D-CRT were similar (I-IMRT vs 3D-CRT; *P* = .016, I-IMRT vs F-IMRT; *P* = .01, and 3D-CRT vs F-IMRT; *P* = .605). No significant differences in V30, V40, and V50 were found among any of the techniques (Table 3).

We also compared the volume and D_mean_ of ICS 1 to 3 for each technique. For all 3 techniques, the median D_mean_ was highest in ICS 3, followed by ICS 2; ICS 1 was lowest. The median mean volumes of ICS 1 to 3 were 2.2, 2.8, and 2.7 cm^3^, respectively; the ICS1 had the lowest volume (Table 4).

**Table 4:**
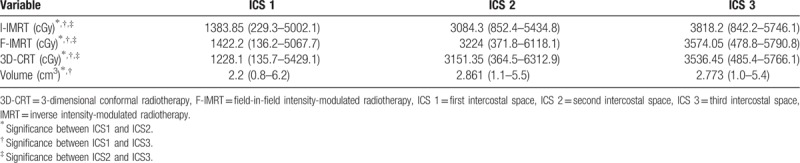
Comparison of the 3 intercostal spaces for each technique.

Table 5 shows the subgroup analysis. For every technique, the patients were separated according to whether they received a SIB (n = 74) or WBRT alone (n = 14) for the analysis. The results showed that the tumor bed boost did not increase the incidental dose to the IMN.

**Table 5:**
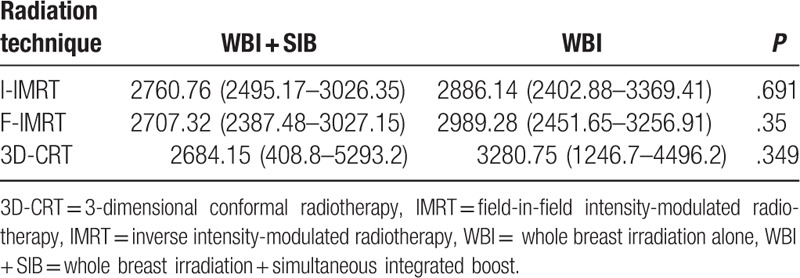
Comparison of the 3 intercostal spaces for each technique (cGy).

Additionally, after analyzing our data further, we noted that 10 of the 84 patients attained a mean dose of over 45 Gy for the IMN_total_. Among the 10 patients, 7 were subjected to 3D-CRT and F-IMRT to attain the adequate dose, 2 patients were subjected to all the 3 techniques, and 1 patient was subjected to 3D-CRT alone.

## 4 Discussion

Arora et al^[15]^ separated ICS 1 to 3 and ICS 1 to 5 for analysis after modified radical mastectomy (MRM) alone. The D_mean_ of the IMN doses obtained for 3D-CRT were 3049 cGy with ICS 1 to 3 and 2498 cGy with ICS 1 to 5. Because most metastatic internal mammary nodes are located in ICS 1 to 3^[16]^ and most guidelines recommend delineating ICS 1 to 3,^[17–20]^ our study evaluated ICS 1 to 3 alone. The median D_mean__total_ doses were 2740.2 cGy (I-IMRT), 2973.9 cGy (F-IMRT), and 2951.4 cGy (3D-CRT) for ICS 1 to 3, and all 3 techniques failed to attain the prescription dose. Moreover, we separated ICS 1 to 3 for analysis and found that all 3 ICS failed to attain an adequate dose. A dose of 45 to 50 Gy is effective for treating subclinical disease. However, Withers et al^[21]^ proposed that a dose of 30 Gy could lead to moderate regional control using a shallow dose–response curve. Another study^[22]^ reported that a microscopic tumor in the ovary or bladder could be sterilized with a relatively low dose (10–30 Gy). Furthermore, in the field of nonsmall cell lung cancer, a mean dose lower than 40 Gy controlled the subclinical disease satisfactorily for the clinically noninvolved mediastinum.^[23,24]^ Similarly, another study^[25]^ analyzed the incidental dose to the axilla at 3 levels with IMRT, 3D-CRT, and standard tangents (ST). The D_mean_ obtained with IMRT, 3D-CRT, and ST were 39, 40, and 43.5 Gy for level I; 35, 36, and 32.5 Gy for level II; and 25.5, 26.5, and 20.5 Gy for level III, respectively. The Z0011 trial^[26]^ showed that the use of sentinel lymph node dissection (SLND) alone and axillary lymph node dissection (ALND) resulted in similar survival outcomes when the patients received tangential whole breast irradiation after BCS. Recently, another study by Kanyilmaz et al^[27]^ compared MRM and BCS. All patients received ipsilateral supraclavicular fossa with a single anterior field (or combined anterior and posterior field). The incidental D_mean_ to the IMN of ICS 1 to 3 were 2670 cGy (BCS) and 3460 cGy (MRM) with 3D-CRT, and the MRM also received a higher dose. Furthermore, after a median follow-up time of 38 months, no case of IMN relapse occurred among the 384 patients, and only 4 patients experienced breast cancer recurrence.

Recently, several large retrospective studies demonstrated that BCS was more effective for early breast cancer than MRM. Agarwal et al^[28]^ retrospectively analyzed 132,149 patients and found that patients who underwent BCS had a higher breast cancer-specific survival rate for early-stage invasive ductal carcinoma than patients treated with mastectomy alone or mastectomy with radiation. A population-based study by Van Maaren et al^[29]^ found that breast-conserving surgery plus radiotherapy was associated with improved 10-year OS compared with mastectomy. The improved survival for BCS may be the result of differences related to reduced injury and adjuvant therapy, such as chemotherapy, target therapy, radiation delivery, or incidental IMN. This finding warrants further investigation. Consequently, in combination with postsurgery chemotherapy, endocrine therapy, and targeted therapy, the effect of preventive low-dose radiotherapy for early breast cancer needs further follow-up or animal experiments for confirmation.

Kanyilmaz et al^[27]^ also analyzed several factors that might influence the D_mean_ of the IMN. The authors found that an advanced T stage, advanced N stage, and type of surgery (MRM) all increased the D_mean_ of the IMN. This was absent in our study. Nevertheless, our study adopted an additional 2 IMRT techniques that were current radiotherapy trends due to their favorable dose coverages and lower doses to organs at risk (OARs).^[30–33]^ We also evaluated the Vx. Table 2 showed that the median V50 of IMN_total_ by the 3 techniques (I-IMRT, F-IMRT, and 3D-CRT) were 16.48%, 19.17%, and 20.11%, respectively. Similarly, the other parameters of Vx for IMN_total_, ICS 1, ICS 2, and ICS 3, did not achieve an adequate volume. We noted that most of the parameters were lowest with I-IMRT, which might be the result of improved homogeneity and conformity for I-IMRT.

**Figure 1:**
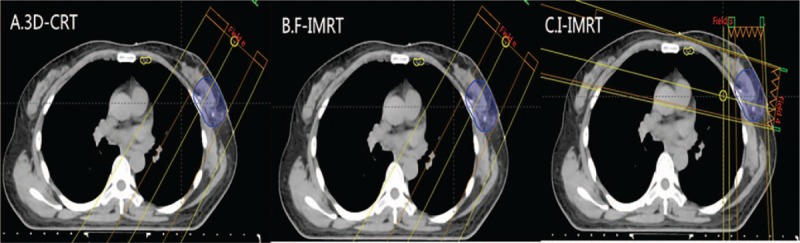
Fields of tumor bed boost of three techniques. (A) Technique of 3D-CRT for tumor boost. (B) Technique of I-IMRT for tumor boost. (C) Technique of F-IMRT for tumor boost. 3D-CRT = 3-dimensional conformal radiotherapy, F-IMRT = field-in-field intensity-modulated radiotherapy, I-IMRT = inverse intensity-modulated radiotherapy.

A similar study used the 3D-CRT technique to examine ICS 1 to 3. Sapienza et al^[34]^ calculated the mean dose to the uninvolved IMN which was 1851 cGy with BCS. The study also irradiated the SCF region according to N stage with a single anterior field (or combined anterior and posterior fields). The results showed that the addition of the FSC field did not increase the mean IMN dose (31.54 vs 29.15 Gy, respectively; *P* = .3766). Therefore, although the SCF region was added, the dose of IMN still failed to be adequate. The authors also found that a boost to the tumor bed did not increase the D_mean_ of IMN, which was consistent with the results of our study. Instead of evaluating three techniques, their study compared 3 surgical styles. The results showed that the D_mean_ to the IMN was highest with MRM and MRM with immediate reconstruction (MRM+R) and lowest with BCS. Consequently, no influence was found on the IMN incidental dose regardless of the radiation technique and surgical style used and whether a tumor bed boost was performed. Further analysis showed that the doses delivered by I-IMRT were higher than the doses delivered by 3D-CRT and F-IMRT in the ICS 3, which might be related to the tumor bed boosts. The tumor beds located in ICS 3 are accounted for 69% of the total tumor beds in our study. Compared with 3D-CRT and F-IMRT, which use an anterior electron beam for the tumor bed boost, I-IMRT consists of 2 photon beams; the first beam is anterior, and the second beam is lean and includes a partial IMN (Fig. 1).

Surgical studies have reported that the incidence of positive internal mammary nodes is highest in the ICS 2 (19%), followed by ICS 3 (17%), then the ICS 1 (16%), ICS 4 (6%), and ICS 5 (2%).^[35]^ Another study also showed that all positive internal mammary sentinel lymph nodes were in the ICS 2 (61.1%, 11/18) and the ICS 3 (38.9%, 7/18).^[11]^ But even without IMN radiotherapy, the recurrence rate of IMN was no more than 1.5% after systematic therapy.^[36]^ In this study, we found that the ICS 2 and ICS 3 receive a higher incidental dose than ICS1 for all of these 3 techniques. We could suppose that the lower recurrence rate of IMN after WBRT may be related to the above result. With a relative high incidence of positive internal mammary nodes in the ICS 1, the incidental dose was inadequate in our study. Therefore, irradiation in this area cannot be avoided; the IMN target must be specifically included for the patient if IMN must be irradiated. For ICS 2, an appropriate median D_mean_ of 30 Gy is under the dose range of 45 to 50 Gy regularly used for irradiation of breast cancer. However, the addition of valid chemotherapy may achieve adequate control of subclinical disease,^[37]^ especially for ICS 3, which receives a relatively higher median D_mean_ (3818.2 cGy) with I-IMRT. For patients with a high risk of metastasis in ICS 3, the unintended radiation may be practical with I-IMRT. In this case, the unnecessary irradiation, which may cause toxicity to the heart, especially for breast cancer patients in the left breast, can be avoided. Additionally, we note that the D_mean_ of IMN_total_ can achieve an adequate dose in some patients. For this group of patients, the challenge is to analyze the factors that contribute to this adequate dose. A precise selection of the group that receives the adequate incidental IM dose may enable avoidance of the IMNI or reduce the irradiation target volume to reduce the potential toxicity. Consequently, further studies are needed.

Our study also has limitations. Because the patients we selected received breast radiotherapy previously, delineation of the tumor bed and CTV_breast_ were not performed by the same oncologist although the same guideline was applied, differences may exist among the oncologists. Nevertheless, the results are convincing.

## 5 Conclusions

In conclusion, we present an early dataset of BCS patients evaluated using 3 techniques with comparisons of the incidental doses to the IMN between the 3 techniques. In our study, none of the techniques attained an adequate dose to cure subclinical disease. Although a therapeutic dose level of 45 to 50 Gy is recommended, the IMN relapse rate is low even though the IMN is typically excluded from the planning target.^[12]^ Therefore, with the development of systematic therapy, further studies are needed to determine whether the incidental dose can meet the clinical requirement. Based on our data, avoiding IMNI using any of the 3 techniques during WBRT is risky when the IMN contains metastasis or when there is a high risk of metastasis. Therefore, IMNI should not be avoided as indicated, and changes of radiotherapy technique cannot make IMN free from radiotherapy.

## Author contributions

**Data curation:** Yuanfang Song, Ting Yu, Min Xu.

**Formal analysis:** Yuanfang Song.

**Investigation:** Qian Shao.

**Methodology:** Tao Sun, Pengfei Qiu.

**Supervision:** Jianbin Li.

**Writing – original draft:** Yuanfang Song.

**Writing – review & editing:** Wei Wang, Jianbin Li.
